# Differences in Mechanical and Physicochemical Properties of Several PTFE Membranes Used in Guided Bone Regeneration

**DOI:** 10.3390/ma16030904

**Published:** 2023-01-17

**Authors:** Syed Saad Bin Qasim, Adel A. Al-Asfour, Moosa Abuzayeda, Ahmed M. Mohamed, Branko Trajkovski, Colin Alexander Murray, Gregor-Georg Zafiropoulos

**Affiliations:** 1 Department of Bioclinical Sciences, Faculty of Dentistry, Kuwait University, Safat 13110, Kuwait; 2 Department of Surgical Sciences, Faculty of Dentistry, Kuwait University, Safat 13110, Kuwait; 3 Department of Prosthodontics, College of Dentistry, MBR University, Dubai P.O. Box 505055, United Arab Emirates; 4 Department of Chemistry, Faculty of Science, Kuwait University, Safat 13060, Kuwait; 5 Faculty of Dentistry, Kuwait University, Safat 13110, Kuwait; 6 Department of Preventive and Restorative Dentistry, University of Sharjah, Sharjah P.O. Box 27272, United Arab Emirates

**Keywords:** guided bone regeneration/GBR, physicochemical properties, PTFE membranes, computerized tomography/CT, micro-CT, nano-CT, porosity

## Abstract

Non-resorbable PTFE membranes are frequently used in dental-guided bone regeneration (GBR). However, there is a lack of detailed comparative studies that define variations among commonly used PTFE membranes in daily dental clinical practice. The aim of this study was to examine differences in physicochemical and mechanical properties of several recent commercial PTFE membranes for dental GBR (Cytoplast^TM^ TXT-200, permamem^®^, NeoGen^®^, Surgitime, OsseoGuard^®^-TXT, OsseoGuard^®^-NTXT). Such differences have been rarely recorded so far, which might be a reason for the varied clinical results. For that reason, we analyzed their surface architecture, chemical composition, tensile strength, Young’s modulus, wettability, roughness, density, thickness and porosity. SEM revealed different microarchitectures among the non-textured membranes; the textured ones had hexagonal indentations and XPS indicated an identical spectral portfolio in all membranes. NeoGen^®^ was determined to be the strongest and OsseoGuard^®^-TXT was the most elastic. Wettability and roughness were highest for Surgitime but lowest for OsseoGuard^®^-NTXT. Furthermore, permamem^®^ was the thinnest and NeoGen^®^ was identified as the thickest investigated GBR membrane. The defect volumes and defect volume ratio (%) varied significantly, indicating that permamem^®^ had the least imperfect structure, followed by NeoGen^®^ and then Cytoplast ^TM^ TXT-200. These differences may potentially affect the clinical outcomes of dental GBR procedures.

## 1. Introduction

Guided bone regeneration (GBR) is considered an important method applied during the reconstruction of alveolar bone for successful dental implant placement. The biological basis for GBR involves assisting the bone growth requirement by establishing a stable immobile base to facilitate the release of growth factors and by preserving the blood supply in areas of dentoalveolar bone defects. GBR promotes bone formation by protecting against an invasion of competing non-osteogenic tissues at the site of bone deficiency. [1,2]. Guided bone regeneration (GBR) is based upon the concept that barriers such as surgical membranes should be used as an essential component of the treatment to exclude rapidly proliferating soft tissue. Thereby, this helps promote angiogenesis and osteogenic cell migration from the defect periphery towards the center, aiding in the creation of well-vascularized granulated tissue, resulting in appropriate osseous healing [1,3,4,5]. Resorbable membranes are frequently used for GBR due to their hydrophilicity and manageability. However, they are often incapable of maintaining the appropriate space needed to cover larger bone defects or dental extraction sockets [6,7]. Non-resorbable polytetrafluoroethylene (PTFE) membranes were designed for use in GBR and their primary clinical advantage is they are capable of retaining their structural integrity during implantation, thereby simplifying surgical procedures while providing biocompatibility and stiffness for space maintenance at the bone defect site. Furthermore, PTFE membranes are superior to resorbable membranes in their ability to maintain the space required for cell occlusion, thereby simplifying surgical procedures through minimizing flap reflection in order to preserve keratinized gingival tissues [4,6,8].

Nevertheless, a complete characterization of each barrier membrane is still necessary [1,3,4,6,8,9]. The first generation of PTFE membranes was fabricated as expandable PTFE (e-PTFE) [2,4,8,9]. Subsequently, high-density polytetrafluoroethylene-based membranes (d-PTFE) have been manufactured from 100% pure medical-grade biologically inert PTFE [10,11,12]. As already reported, their density and small pore size should help in protecting the underlying bone graft from bacterial and soft tissue penetration [1,3,13,14]. Furthermore, additional surface texturing of PTFE membranes contributes towards an increase of stability through cell adhesion [15,16,17]. 

Although previous investigations have reported the ideal characteristics of PTFE membranes, studies comparing their properties are sparse [5,7,11,18,19]. However, various manufacturers claim distinct and questionable properties and have used quite confusing terminology. Therefore, the aim of this study was to investigate the various physicochemical and mechanical properties of several commercially available PTFE membranes to clarify their properties for potential advantages and disadvantages in clinical use.

## 2. Materials and Methods

### 2.1. Membrane Specifications

Six commercially available non-Titanium-reinforced PTFE membranes, which are regularly used in GBR approaches, were examined (Table 1). 

### 2.2. Scanning Electron Microscopy (SEM)

For SEM, specimens were cut at 5 × 5 mm from the membranes and mounted on aluminum stubs. These were sputter coated under vacuum with gold (JFC-1600; Jeol, Akishima, Japan) and viewed through the SEM (JSM-In Touch Scope 200; Jeol, Akishima, Japan) with a voltage range of 15 kV, working distance of 10.8 mm at ×50, ×100 and ×500 magnification. Images were acquired from the top and the bottom membrane surfaces to evaluate discrepancies. For specimens with different morphology, images are shown as insets in the results section.

### 2.3. Surface Chemical Structure and Composition—X-ray Photoelectron Spectroscopy (XPS)

The elemental surface chemical composition of membranes was analyzed by x-ray photoelectron spectroscopy (XPS). XPS data was collected from an ESCA Lab 250Xi (Thermo scientific, Waltham, MA, USA) instrument equipped with a monochromator excitation source of Al Kα (1486.5 eV and spot size 850 mm), argon ion gun and flood gun neuralization sources. The instrument was calibrated with a standard holder containing gold, copper and silver. The binding energies of Au4f7/2, Cu2p3/2 and Ag3d5/2 were 84.0, 932.6 and 368.2 eV after the surface was cleaned with an argon ion gun. The spectra binding energies corrected concerning adventitious carbon of C1s at 284.6 eV. To avoid and reduce impurities contamination, a small piece of each specimen of dimensions 5 mm × 5 mm was cut from the specimen immediately after unpackaging and loaded to the instrument. The specimens were fixed on a sample holder with an adhesive double-side carbon tab and maintained in the preparation chamber until the vacuum reached 10^−7^ mbar, then transferred to the analysis chamber for irradiation at UHV of 10^−9^ mbar. Escaped electrons after irradiation were collected with a spectrometer and analyzed according to their kinetic energies. The produced spectra were processed and deconvoluted with Avantage software (v5.956; Thermo Scientific, Waltham, MA, USA). The survey spectra were collected using Constant Analyzer Energy (CAE) scan mode in the wide range of 0–1300 eV at 150 eV pass energy, 50 ms dwell time and 1 eV step size. High-resolution narrow scans were performed at 20 eV pass energy, 50 ms dwell time and 0.1 eV step size for a different number of scans depending on peak intensity. The narrow scan ranged for core-level of C1s and F1s from 282–298 eV and 680–698 eV, respectively. 

### 2.4. Mechanical Properties

Membranes were tested using a tensile testing machine (Instron, ElectroPuls™ E3000; Norwood, MA, USA) equipped with a load cell of 1 kN. Barrier membranes were cut into cylindrical specimens with dimensions of 15 mm height, 5 mm diameter. A sectioned custom-made acrylic mould was prepared, and the middle 5 mm protected with wax and loose ends embedded in cold cure self-curing acrylic resin [5]. Such specimens are prepared to avoid the membranes to slip away from the clamps. The resin embedded specimen ends were then held by Instron clamps. The central square portion had an area of 25 mm^2^ and free of any acrylic or wax. Tests were conducted in quadruplicates. From the obtained strength–strain curves, the point at which samples demonstrated deformation were used to calculate the ultimate tensile strength (UTS; MPa) and strain (%), whilst the initial linear graph was used to calculate the Young’s Modulus (E; i.e., tensile strength).

### 2.5. Physical Properties

#### 2.5.1. Wettability

The surface wettability of the highly segregated membranes was evaluated by polar liquid (distilled water) contact angle (CA) measurements. A drop of distilled water (3 μL) was added on membrane surfaces at 20 °C. The contact angle was measured by the sessile drop method using a drop shape analyzer (DSA100B; Krüss, Hamburg, Germany). The Krüss drop shape analysis program (ADVANCE 1.7.2.1; Krüss, Hamburg, Germany), determined the contact angle in two steps. In the first step, the drop image was subjected to a gray level analysis. The result was an optically determined contour line around the phase boundary in the drop image. In the second step, this drop contour was calculated mathematically. The contact angle was obtained from the angle between the drop contour function and sample surface. To minimize experimental error, three readings were performed for each specimen and their average value reported. Range of results was from 0 to 180°. To determine the dynamics of CA change with time, the shape of the liquid droplet was recorded by the camera starting from second zero until 5 min with a resolution of 0.01° and accuracy of 0.1°.

#### 2.5.2. Surface Roughness

The surface roughness of PTFE membranes was quantitatively characterized using an optical profilometer LEICA DCM 8 (Leica Microsystems CMS GmbH, Wetzlar, Germany). The objective lens used for scanning the specimens was at 10× magnification. Stationary type of roughness measurements were assessed in air without any sample preparation [20]. Specimens were placed on a clean glass slide before imaging. The aerial surface roughness parameters, including the arithmetic average height of the surface (Ra; Robust Guassian filter 0.25 mm) and root mean square (Rq) gradient, were measured according to ISO25178 (ISO 2012) over a sampling length of 500 µm. Three readings were taken from the surface, expressed as µm. Due to the different structure, measurements of Cytoplast^TM^ TXT-200 and OsseoGuard^®^-TXT were acquired by taking points from hexagonal indentations and the non-textured areas between them.

#### 2.5.3. Density

The membrane apparent density is meant to correlate inversely with the degree of expansion. The mass per area was calculated by multiplying the length to width (10 × 10 mm) of the specimens. Subsequently, the density of the membranes was calculated by dividing the mass per area over membrane thickness in g/dm^3^.

#### 2.5.4. Nano-Computerized Tomography

PTFE membrane thickness and porosity was examined using a nano-CT (GE Phoenix Nanotom^®^ M; GE Sensing & Inspection Technologies GmbH, Hürth, Germany), equipped with a nano-focus tube and fitted with an air cushioned turntable to minimise friction which aided acquisition of a series of X-rays as samples revolved at 360°. The beam voltage was set at 100 kV, amperage at 100 μA with a final isotropic resolution of 6.67µm per voxel. A 0.5 mm aluminium filter was used. Each membrane was scanned for 1.45 h, which resulted in acquiring 2000 images. The acquired data were used to fabricate a digital volumetric reconstruction via a calculation algorithm using VG studio Max. 3.2.5 software (Volume Graphics GmbH, Heidelberg, Germany). Following reconstruction, the area to determine the pores was manually adjusted. The porosity inclusion option VGEasy was used to conduct pore analysis. Analysis parameters were adjusted accordingly with respect to contrast, surface distance and filters, and adjusted for probability thresholds. Results of the thickness measurements were expressed in micro-meters (μm). Porosity analysis revealed for each material and defect volume (μm^3^) as well as the % defect ratio.

### 2.6. Statistical Analysis

All tests were performed 3 times and mean values were used. To compare outcome variables, analyses of variance (ANOVAs) were used, all of which were measured on continuous scales, among the six membranes. Unpaired *t*-tests were used to compare measurements between membranes with non-textured versus those with textured surfaces (two groups) and also to compare Cytoplast^TM^ TXT-200 versus OsseoGuard^®^-TXT membranes specifically. For both the ANOVAs and *t*-tests, variables whose data did not meet the data analysis requirements of these methods (parametric data distribution and equal variability) were first transformed to log scale values to enable analyses. The continuous variables of material volume, defect volume, defect volume ratio (%) and thickness were compared among the six membranes with non-parametric Kruskal–Wallis H tests (total n = 59, degrees of freedom = 5 in all cases) followed by Dunn’s post hoc tests with Bonferroni’s correction for multiple comparisons. These four datasets did not meet the conditions for parametric data analysis. Mann–Whitney U (Wilcoxon rank–sum) tests were used to compare measurements between textured and non-textured membranes, and to compare the OsseoGuard^®^-TXT versus OsseoGuard^®^-NTXT membranes specifically. The criterion for significance was *p* = 0.05 for the Kruskal–Wallis H tests and the Mann–Whitney U tests; a Bonferroni’s multi-comparison corrected significance criterion of *p* < 0.33 was applied to the Dunn’s post hoc tests.

## 3. Results

### 3.1. Scanning Electron Microscopy (SEM)

**Figure 1:**
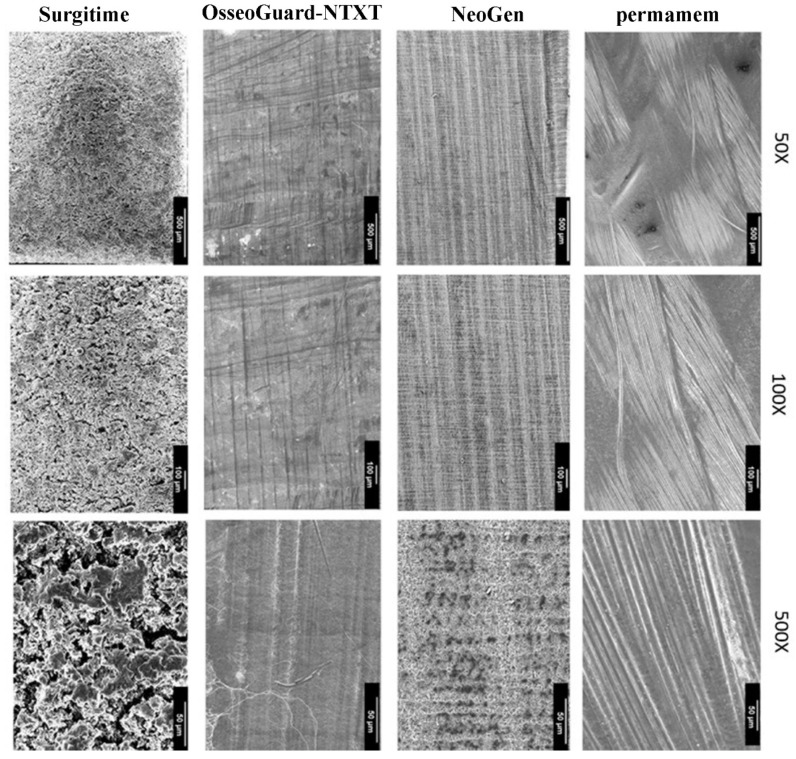
SEM images of the soft tissue-facing site of the non-textured PTFE.

SEM analysis was undertaken to investigate the membrane microstructures (Table 2). A multidirectional and heterogenous fibrillar orientation pattern with tightly packed non-porous microstructure and areas with and without macro-waviness was shown for permamem^®^ (Figure 1). OsseoGuard^®^-NTXT had bi-directional and fibrillar microstructure, which, contrary to the manufacturer claims, revealed no pores, even at higher magnification. Furthermore, the dual layer membrane NeoGen^®^ showed a monodirectional fibril orientation with a porous microstructure that was visible on higher magnification. These pores had an approximate size of 1 to 2 µm. Surgitime showed a non-fibrillar heterogenous morphology and irregular semi-open microarchitecture with pores ranging from 10 to 25 µm.

**Figure 2:**
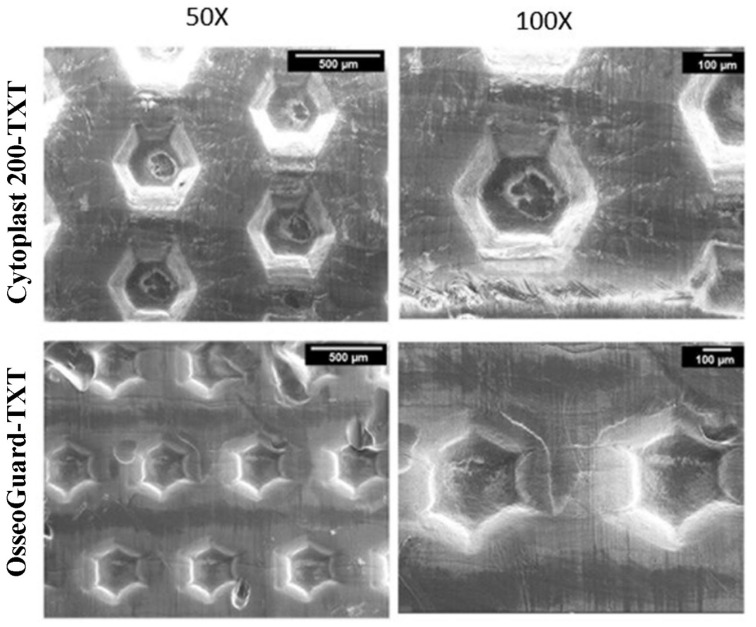
SEM images of the textured PTFE membranes.

Both Cytoplast^TM^ TXT-200 and OsseoGuard^®^-TXT displayed a non-fibrillar structure with evenly distributed hexagonal-shaped indentations (Figure 2). These indentations had a 500 µm width with 250 µm spacing in between. The depth of these indentations was less than the core thickness of the membranes, both were very similar in surface features and no pores were seen.

**Figure 3:**
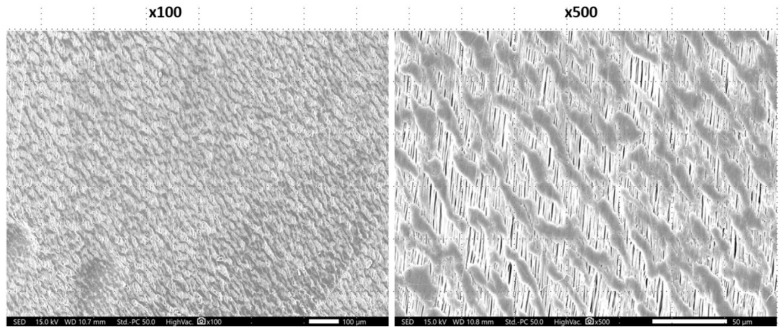
NeoGen^®^ membrane. SEM images of the bone-facing site.

The bone-facing surfaces of all non-textured membranes had similar characteristics with the soft tissue-facing sites, and textured membranes had similar characteristics with the flat areas between the indentations of the soft tissue-facing sites. Only NeoGen^®^ was an exception, having a bone-facing site characterized by unidirectional orientation of the fibrils with nodes appearing in oblique direction to the fibrils (Figure 3).

### 3.2. X-ray Photoelectron Spectroscopy (XPS)

**Figure 4:**
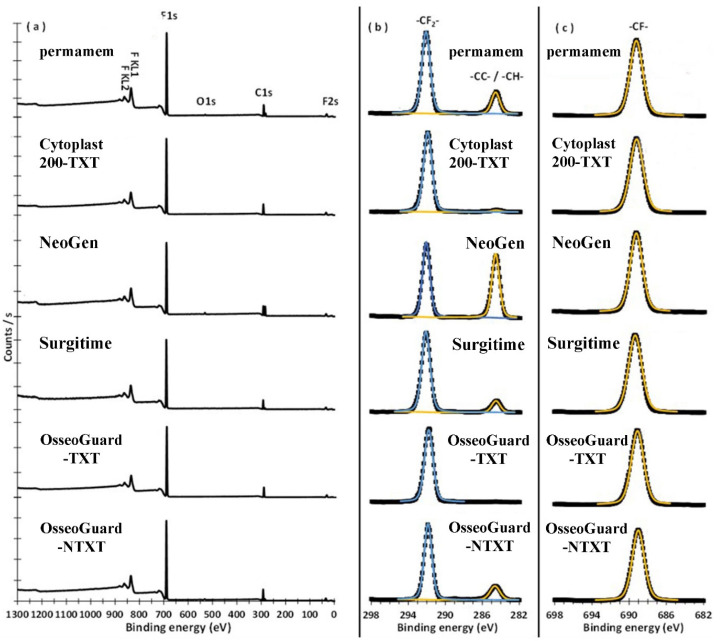
XPS spectra. (**a**) survey, (**b**) C1s, (**c**) F1s regions; TXT: textured; NTXT: non textured.

The obtained results are in Table 2 and Figure 4. The wide scan in the range of 0–1300 eV of survey spectrum for permamem^®^ (Figure 4a) identified the presence of four XPS peaks for F2s, F1s, O1s and C1s. This reveals that its surface contains Carbon (C), Fluorine (F) and a minute percentage of Oxygen (O) atoms. The high-resolution deconvolution peaks for C1s identified the existence of two states of carbons assigned to adventitious carbon (-CC-/-CH-) and carbon-fluorine bonds (-CF_2_-) measured at 284.63 and 292.05 eV, respectively (Figure 4b). F1s spectrum of permamem^®^ revealed that only one state of fluorine, measured at 689.25 eV, corresponded to the C-F bond present on the membrane surface (Figure 4c). The atomic percentage ratio of C1s peak of (-CF_2_-) to the F1s peak was about 0.5, confirming that permamem^®^ is made of polytetrafluoroethylene (CF_2_-CF_2_-)n. The survey spectra of NeoGen^®^ and OsseoGuard^®^-NTXT showed the same elemental structure of C, F, and O as permamem^®^ (Figure 4a). Conversely, the survey spectra for Surgitime, Cytoplast^TM^ TXT-200 and OsseoGuard^®^-TXT confirmed that only C and F were the main structure of the surface of these membranes. The XPS spectra regions for C1s demonstrated that the surface of all membranes contained adventitious carbon (-CC-/CH-) with varying percentages and, except OsseoGuard^®^-TXT, the majority of membrane surfaces were carbon-fluorine bonds (Figure 4b). These carbon-fluorine bonds (-CF_2_-) were measured at 291.96, 292.16, 291.85, 291.86 and 292.09, eV on the surface of Cytoplast^TM^ TXT-200, Surgitime, OsseoGuard^®^-TXT, OsseoGuard^®^-NTXT and NeoGen^®^. For all membranes, there was one state for F on the surface corresponding to the -CF- structure measured in the range of 689.02–689.37 eV (Table 2). Moreover, the presence of adventitious carbon and/or adsorbed hydroxide group (OH) and water may be from exposure of specimens to air during the handling. Specimens with the highest percentage of adventitious carbon identified the presence of adsorbed -OH/H_2_O on their surfaces. The membranes of NeoGen^®^, permamem^®^ and OsseoGuard^®^-NTXT showed -OH/H_2_O adsorbed on the surface and the highest amount of adventitious carbon of 22.2, 7.93 and 7.37%, respectively.

### 3.3. Mechanical and Physical Properties

ANOVAs revealed significant main effects of membrane type on the mechanical properties of ultimate tensile strength (UTS) and Young´s Modulus (E), but not on strain (Table 3). The results suggested statistically significant differences between the six membranes for UTS and E. However, no significant differences were observed between the membranes for strain. Both UTS and E values were found to be highest for the NeoGen^®^ membrane and lowest for the OsseoGuard^®^-TXT membrane.

Regarding physical properties, surface roughness and wettability varied significantly among the six membranes. While Surgitime had the highest wettability values, OsseoGuard^®^-NTXT had the lowest values. Furthermore, permamem^®^, NeoGen^®^ and OsseoGuard^®^-TXT had similar results to each other, and Cytoplast^TM^ TXT-200 demonstrated a lower wettability compared to these membranes (Table 3). Surgitime membrane had by far the highest roughness values, with a mean of 32 μm. This contrasted with a mean of less than 1 μm for OsseoGuard^®^-NTXT, which had the lowest mean roughness value. Then, permamem^®^ was rougher than OsseoGuard^®^-NTXT, followed by NeoGen^®^ and the two textured membranes (Cytoplast^TM^ TXT-200 and OsseoGuard^®^-TXT) (Table 3).

There was only weak evidence of a difference in density between membranes. More specifically, NeoGen^®^ showed the lowest (1.27 g/cm^3^) and Surgitime showed the highest density (1.67 g/cm^3^).

As shown in Table 4, membranes with non-textured surfaces (combined group of four membranes, namely permamem^®^, NeoGen^®^, Surgitime and OsseoGuard^®^-NTXT) had significantly greater mean E and wettability values than membranes with textured surfaces (combined group of Cytoplast^TM^ TXT-200 and OsseoGuard^®^. TXT). None of the other mechanical and physical property characteristics differed significantly between non-textured and texture membranes (Table 4). Comparing the two textured membranes to each other (Cytoplast^TM^ TXT-200 and OsseoGuard^®^-TXT), OsseoGuard^®^-TXT had significantly greater strain and wettability values than Cytoplast^TM^ TXT-200 (Table 4). Notably the mean strain value obtained for OsseoGuard^®^-TXT was more than three-fold compared to Cytoplast^TM^ TXT-200. Statistically similar mean UTS, E, surface roughness and density values were obtained for these two membranes.

**Figure 5:**
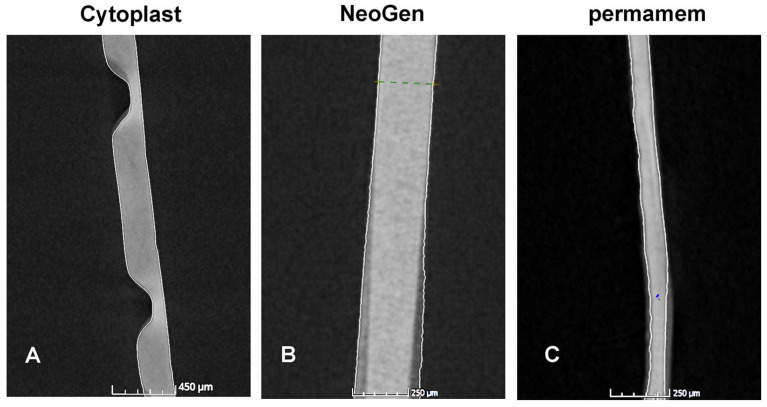
Nano-CT examination of the tested membranes. Representative axial sections from textured and non-textured membranes. (**A**) Cytoplast^TM^ TXT-200 textured membrane with hexagonal shaped indentations on the soft-tissue site and flap surface in the bone tissue site; (**B**) NeoGen^®^: the thickest non-textured membrane; (**C**) permamem^®^: the thinnest non-textured membrane.

**Figure 6:**
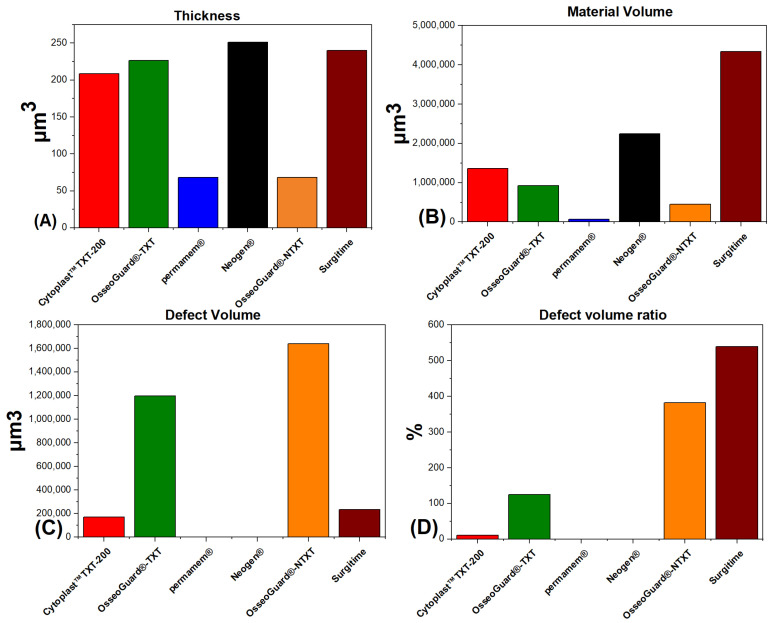
Diagrammatic representation of the nano-CT obtained results. (**A**) Thickness; (**B**) Material Volime; (**C**) Defect Volume; (**D**) Defect Volume Ratio.

Regarding the nano-CT results, NeoGen^®^ was the thickest while permamem^®^ was the thinnest among the non-textured membranes (Figure 5) and the descriptive statistics for each of the six compared membranes are reported in Figure 6. Descriptive statistics for investigated PTFE membranes for which selective inter-group comparisons were planned to examine surface texture as a factor, including combined groups of all textured membranes and all non-textured membranes, are reported in Table 5.

The material volumes of the six membranes varied significantly (Kruskal–Wallis H test: χ^2^(5) = 51.64, *p* < 0.001, effect size 0.88; mean rank scores of 5 for permamem^®^, 34 for Cytoplast^TM^ TXT-200, 41.7 for NeoGen^®^, 54.5 for Surgitime, 26.2 for OsseoGuard^®^-TXT, 16.1 for OsseoGuard^®^-NTXT). Post hoc Dunn’s tests using a Bonferroni corrected alpha of 0.0033 indicated that the mean rank of permamem^®^ differed from the mean ranks of the Cytoplast^TM^ TXT-200, NeoGen^®^, and Surgitime; the NeoGen^®^ mean rank additionally differed from that of the OsseoGuard^®^-NTXT, and the Surgitime mean rank differed from that of the OsseoGuard^®^-TXT.

Defect volumes observed for the six membranes varied significantly (Kruskal–Wallis H test: χ^2^(5) = 41.62, *p* < 0.001, effect size 0.7; mean rank scores of 8.67 for permamem^®^, 24.8 for Cytoplast^TM^ TXT-200, 17 for NeoGen^®^, 32.8 for Surgitime, 44.5 for OsseoGuard^®^-TXT, 50.1 for OsseoGuard^®^-NTXT). Post hoc Dunn’s tests (Bonferroni corrected alpha = 0.0033) results indicated that the mean ranks of the following pairs of membranes were significantly different: permamem^®^ versus Surgitime, OsseoGuard^®^-TXT, and OsseoGuard^®^-NTXT; Cytoplast^TM^ TXT-200 versus OsseoGuard^®^-NTXT; NeoGen^®^ versus OsseoGuard^®^-TXT and NeoGen^®^ versus OsseoGuard^®^-NTXT.

Percentage defect volume ratios varied significantly among the six membranes (χ^2^(5) = 46.15, *p* < 0.001, effect size = 0.79; mean rank scores of 8.67 for permamem^®^, 31.6 for Cytoplast^TM^ TXT-200, 17.7 for NeoGen^®^, 22.5 for Surgitime, 44.1 for OsseoGuard^®^-TXT, 53.3 for OsseoGuard^®^-NTXT). Post hoc Dunn’s tests (Bonferroni corrected alpha = 0.0033) indicated that the mean ranks of the following pairs of groups were significantly different: permamem^®^ versus OsseoGuard^®^-TXT OsseoGuard^®^-NTXT; NeoGen^®^ versus OsseoGuard^®^-TXT OsseoGuard^®^-NTXT; Surgitime versus OsseoGuard^®^-NTXT. Additionally, the difference between the mean rank of the Cytoplast^TM^ TXT-200 identified near significant differences with the mean ranks of the OsseoGuard^®^-NTXT (*p* = 0.0035) and permamem^®^ (*p* = 0.0047). These comparisons became insignificant following application of Bonferroni corrections for multiple comparisons.

Thicknesses were found to vary significantly among the six membranes (χ^2^(5) = 52.62, *p* < 0.001, effect size = 0.9; mean rank scores of 5.0 for permamem^®^, 17.6 for Cytoplast^®^ TXT-200, 51.2 for NeoGen^®^, 46.8 for Surgitime, 35.5 for OsseoGuard^®^-TXT, and 21.4 for OsseoGuard^®^-NTXT). Post hoc Dunn’s tests (Bonferroni corrected alpha = 0.0033) indicated that the mean ranks of the following pairs of membranes were significantly different: permamem^®^ versus NeoGen^®^, Surgitime, and OsseoGuard^®^-TXT; Cytoplast^TM^ TXT-200 versus NeoGen^®^ and Surgitime; NeoGen^®^ versus OsseoGuard^®^-NTXT; and Surgitime versus OsseoGuard^®^-NTXT.

A Mann–Whitney U test indicated that, compared to a combined group of the four non-textured membranes, a combined group of the two textured membranes (Cytoplast^TM^ TXT-200 and OsseoGuard^®^-TXT) had significantly lower defect volume ratios (*p* = 0.01174, z statistic = 2.5199, effect size 0.33). Material volumes (*p* = 0.9808, z statistic 0.024.03, effect size 0.0031), defect volumes (*p* = 0.1364, z statistic = 1.4894, effect size 0.19), and thicknesses (*p* = 0.2727, z statistic = −1.0969, effect size 0.14) were statistically similar between the combined non-textured membranes and the combined textured membranes. 

Comparing OsseoGuard^®^-NTXT to OsseoGuard^®^-TXT it was observed that OsseoGuard^®^-TXT had significantly lower material volumes (*p* = 0.01102, z statistic = 2.5419, effect size 0.57), defect volume ratios (*p* = 0.00444, z statistic = −2.8455, effect size 0.64) and thicknesses (*p* < 0.0001, z statistic = 4.4, effect size 0.84). However, these two membranes had similar defect volumes (*p* = 0.3055, z statistic = −1.0248, effect size 0.23).

The two textured-surface membranes examined, Cytoplast^TM^ TXT-200 and OsseoGuard^®^-TXT, differed significantly in all four outcome variables. Compared to the OsseoGuard^®^-TXT group, Cytoplast^TM^ TXT-200 had significantly greater material volumes (*p* = 0.04507, z statistic = 2.004, effect size 0.45) and defect volumes (*p* = 0.00281, z statistic = −2.9882, effect size 0.67), while having significantly lower defect volume ratios (*p* < 0.00282, z statistic = −2.987, effect size 0.67) and thicknesses (*p* < 0.0001, z statistic = −4.4, effect size 0.84).

## 4. Discussion

The aim of this study was to analyze the mechanical and physicochemical properties of six commercially available PTFE membranes used in dental GBR. Such differences can possibly influence their clinical handling and GBR outcomes. Although the PTFE materials of some of the commercially available membranes we tested is the same in the Titanium-reinforced and in the non-Titanium-reinforced products, we selected the latest PTFE membranes so that we could examine the properties without any influence from the membrane reinforcement. Some of the membranes tested in our study have been reported in the literature and successful clinical outcomes have been recorded. However, there is lack of detailed knowledge in variations of the mechanical and physicochemical properties of commercially available PTFE membranes that can provide appropriate indication-oriented selection, and no comparative study regarding their mechanical properties has been performed [1,2,5,11,18,19,21]. 

Despite their molecular homogeneity, our SEM observations demonstrated that the examined PTFE membranes were morphologically heterogenous with respect to fiber orientation and pattern arrangement. The non-textured membranes, namely OsseoGuard^®^-NTXT, NeoGen^®^, Surgitime and permamem^®^, exhibited highly pronounced morphological heterogeneity. High-magnification images revealed extensive surface aberrations on Surgitime and NeoGen^®^ membranes but relatively smooth surface profiles for permamem^®^ and OsseoGuard^®^-NTXT. High-magnification images of the two investigated textured surface membranes, Cytoplast^TM^ TXT-200 and OsseoGuard^®^-TXT, revealed identical hexagonal indentations that were evenly distributed over the material surfaces which is consistent with previous descriptions [18,19]. These indentations may increase GBR stability by facilitating cell adhesion.

The XPS results revealed explicit surface chemical and elemental compositions of the tested membranes. It was identified that the investigated specimens had an almost identical spectral portfolio, which was confirmed by the data acquired through quantitative analysis. Moreover, the revealed atomic composition was in accordance with previous studies [22,23]. The revelation of carbon, fluorine and oxygen levels identified that the membranes were fully fluorinated [24]. It did not detect any oxygen levels in Surgitime, Cytoplast^TM^ TXT-200 or OsseoGuard^®^-TXT, which might be a result from interference by the porosity in Surgitime and the hexagonal-shaped indentations texture in the others. From the overall spectra lines and the -CF_2_-/F ratio of ≈ 0.5, it can be confirmed that the surface of all six tested membranes consisted of polytetrafluoroethylene (-CF_2_-CF_2_-)_n_. Such inert properties of these PTFE membranes are expected to prevent any adverse type of reactions at the hosting site. Hence, the XPS results indicate that the membranes are indeed bioinert.

PTFE membranes used for GBR should be able to withstand the pressure of the overlying soft tissue and keep its shape to maintain the space for osseous regeneration. Furthermore, they should be easily manipulated plastically without collapsing. High elasticity and flexibility can enable a membrane to be maximally adapted to the defect, thus stabilizing the bone graft. In such cases, a stiff membrane cannot be contoured easily and may impede rehabilitation [5,21]. However, high stiffness, a quality related mainly to membrane thickness, is required for a membrane to enable the surgeon to create and maintain a suitable space for bone regeneration [2,6,7]. Among the membranes examined in this study, OsseoGuard^®^-TXT had the greatest flexibility (i.e., lowest stiffness as indexed by E value), followed in decreasing order by Cytoplast^TM^ TXT-200, permamem^®^, Surgitime, OsseoGuard^®^-NTXT and finally, NeoGen^®^. The relatively high stiffness (high E value) of NeoGen^®^ could be the consequence of the dual-membrane-layer structure of the NeoGen^®^ membrane [7,18,19]. The high flexibility of these membranes, i.e., low stiffness, indicates that these membranes could be easily adapted over the bone defect and also removed after the healing period. The lower E values of the other membranes could be due, at least in part, to the manufacturing process. However, there were also inherent differences in the node and fibril microstructure across the materials that would be expected to affect stiffness [25]. 

When a force is applied under tensile conditions, PTFE fibers can slide freely in the direction of the applied load until fiber breakage occurs. Under tensile conditions, the membranes’ robust behavior might be attributable to the polymers undergoing plastic deformation with time under the strength of a high force load. This behavior should be clinically useful for bone regeneration surgeries. High tensile strength and stability of membranes are required for combined horizontal and vertical bone ridge augmentation, where relatively large defects have to be regenerated and the membrane must be stabilized by being fixed with pins to the surrounding bone [26]. Of the six membranes examined, NeoGen^®^ yielded the highest UTS and OsseoGuard^®^-TXT yielded the lowest one. Relatively lower UTS values were obtained for the remaining materials, in the following decreasing order: OsseoGuard^®^-TXT, OsseoGuard^®^-NTXT, Cytoplast^TM^ TXT-200, Surgitime and permamem^®^. The other four membranes showed intermediate variations in these properties related to their particular levels of stiffness and abilities to undergo plastic deformation under tension. Notwithstanding, our mechanical testing results demonstrated that all six tested PTFE membranes were robust during tensile loading, with no significant differences among them with respect to strain data, and they had the mechanical capacity to withstand surgery-associated forces.

Surface wettability was investigated using the CA technique. All examined membranes displayed hydrophobic properties. This has been attributed to the membrane reticular fiber nodule structure that is formed due to the melt stretching membrane fabrication technology [27]. The high CA values negatively affect protein adsorption, platelet adhesion/activation, blood coagulation and cell bacterial adhesion [24,28,29]. All six tested membranes were found to be hydrophobic. Meanwhile, surface roughness is a key factor in susceptibility to cell and microbial plaque adhesion [30]. Our findings identified that Surgitime had both the highest surface roughness value and the highest wettability value. Converesely, OsseoGuard^®^-NTXT had the lowest values. These observations are consistent with the supposition that material wettability can be enhanced by increasing surface roughness, as predicted by Wenzel’s roughness–wettability relationship model [31]. Furthermore, regarding roughness and wettability, the differences observed in the examined membranes could be explained by the fact that the intrinsic CA is affected by the roughness and physicochemical properties [32]. Thus far, there is no universally accepted optimum roughness level for inhibiting the adhesion of all bacterial species [33].

High density has been reported to correlate with infection prevention in PTFE membranes, prohibiting penetration and subsequent infiltration of the covered osseous defect [16,34]. The obtained results demonstrated that permamem^®^ had the highest density, followed by Surgitime. However, a comparison of density values among all six membranes could not provide any statistical difference. The observed high densities of the tested membranes indicate that all membranes have the benefit of relatively straightforward removal from the augmented/regenerated area. 

Porosity is an important property of GBR membranes and could influence the degree of bone regeneration in the underlying defect. Therefore, it is considered as being closely related to tissue occlusivity through having an influence on the invasion of cells [11,35,36,37,38]. In the present study, nano-computed tomography (nano-CT) was used as a high-resolution cross-sectional imaging technique [39]. We observed that permamem^®^ had the lowest and correlating values between material volume and thickness, as well as defect volume and defect volume ratio, which makes it the thinnest and less imperfect when compared to the other analyzed membranes. Conversely, Surgitime had the highest material volume but NeoGen^®^ was the thickest, as OsseoGuard^®^-NTXT had the highest defect volume and defect volume ratio, which makes it the most imperfect among the investigated membranes. Strangely, the presence of hexagonal shaped indentations in the textured membranes resulted in lower defect volume ratios when compared to the non-textured ones. However, Cytoplast^TM^ TXT-200 had higher material and defect volumes, but much lower defect volume ratio and thickness when compared to OsseoGuard^®^-TXT, which indicates thinner structure into the indentations. 

The limitations of the present study have to be considered. The physicochemical properties were assessed under controlled laboratory conditions, and it is not known if the obtained results could be exactly transferred to clinical practice. Furthermore, the precise relationship between the surface properties of the examined membranes and the adhesion of cells and/or microorganisms on each of them was not investigated. In a recent publication, it has been mentioned that crystallite features and the degree of crystallinity (depending on the fabrication process and thermal history) affect PTFE physicochemical and mechanical properties [17,19].

Although the obtained results indicated that all tested membranes were hydrophobic, the non-textured membranes had higher stiffness and hydrophobicity compared to the textured ones. Nevertheless, the two examined textured membranes had similar surface morphology, they were found to have different wettability and stiffness properties. Cytoplast^TM^ TXT-200 demonstrated lower wettability and higher flexibility (i.e., stiffness) than OsseoGuard^®^-TXT, which probably leads to an easier adaptation of Cytoplast^TM^ TXT-200 over the bone defect.

The tested membranes are widely used in dental GBR surgery. Different non-resorbable and bioresorbable barrier membranes have been developed and their use has been extensively investigated, research is ongoing to develop an ‘ideal’ GBR membrane. The basic characteristics of such membranes should be biocompatibility, cell-occlusiveness, space-making, tissue integration and clinical manageability [8,40,41]. The present study of the physicochemical and mechanical properties of six examined PTFE membranes showed that all membranes were bioinert and, consequently, may favor good tissue integration. Furthermore, all investigated membranes were found to be hydrophobic, which indicates they were occlusive, could protect the augmented bone defect from saliva, serum, protein and other fluids, favor soft tissue interaction and act as barriers to bacteria, thereby minimizing the risk of infection. We could hypothesize that clinically, due to the assessed density, all membranes could be smoothly removed after completion of the tissue healing period. However, the individual observation of the tested materials discovered differences in other physicochemical properties. An ideal PTFE membrane for GBR surgical approaches should have high flexibility, low stiffness, high hydrophobicity and low roughness and be chemically inert. Actual research on functionalization of the PTFE surfaces using recombinant spider silk seems promising and could improve the clinical outcomes of GBR [25].

The obtained results may be of a significant help to the dental surgeon, by the selection of the right type of membrane according to the clinical condition (i.e., shape, size and volume of the bone defect) that has to be regenerated. The observed differences among the membranes could reflect miniscule alterations in their microarchitecture and non-uniformity of their thicknesses. Furthermore, combined clinical and laboratory research should be directed towards optimizing their physical and mechanical properties, as well as bacterial adhesion and penetration, so that they facilitate the surgical approach and aid the clinician´s expectations.

## 5. Conclusions

The obtained results indicate that all PTFE membranes examined in this study were bioinert, hydrophobic and had similar densities. However, other properties like tensile strength, stiffness and porosity varied, which will impact on their application for GBR. The selection of the optimal GBR membrane depends upon the specific surgical approach involved. Further studies of the tested PTFE membranes investigating cellular adhesion as well as a clinical comparison of the specific characteristics are required.

## Figures and Tables

**Table 1:** Specifications of the used membranes as claimed by the manufacturers.

Membranes	Structure	Surface	Manufacturer
permamem^®^	hd-PTFE	NTXT	botiss biomaterials GmbH, Zossen, Germany
Cytoplast^TM^ TXT-200	hd-PTFE	TXT	Osteogenics Biomedical Inc., Lubbock, TX, USA
NeoGen^®^	Dual e-PTFE	NTXT	Neoss Ltd., Harrogate, UK
OsseoGuard^®^-TXT	hd-PTFE	TXT	Zimmer Biomet Dental, Palm Beach Gardens, FL, USA
OsseoGuard^®^-NTXT	hd-PTFE	NTXT	Zimmer Biomet Dental, Palm Beach Gardens, FL, USA
Surgitime	PTFE	NTXT	Bionnovation Biomedical, Bauru, Brazil

hd: high density; TXT: Textured; NTXT: Non-textured.

**Table 2:** Surface characteristics of membranes compared in this study.

Property	Membrane Type					
	permamem^®^	Cytoplast^TM^ TXT-200	NeoGen^®^	Surgitime	OsseoGuard^®^ TXT	OsseoGuard^®^ NTXT
SEM characteristics						
Fibril orientation	multidirectional, heterogenous, fibrillar	hexagonal shaped indentations, non-fibrillar	Monodirectional, fibrillar	heterogenous, non-fibrillar	hexagonal shaped indentations, non-fibrillar s	bi-directional, fibrillar
XPS surface characteristics						
C1s BE, (FWHM), (a%), CB	284.63, (1.06), (7.93), -CC-/-CH-292.05, (1.05), (30.13), -CF_2_-	284.61, (1.28), (0.90), -CC-/-CH-291.96, (1.06), (32.83), -CF_2_-	284.64, (0.96), (22.21), -CC-/-H- 292.09, (0.92), (25.25), -CF_2_-	284.61, (1.07), (4.30), -CC-/-CH-292.16, (1.07), (30.12), -CF_2_-	291.85, (1.09), (33.92), -CF2-	284.63, (1.26), (7.37), -CC-/-CH-291.86, (0.98), (31.14), -CF_2_-
F1s BE, (FWHM), (a%), CB	689.25, (1.62), 61.10), -CF-	689.21, (1.70), (66.27), -CF-	689.26, (1.56), (50.88), -CF-	689.37, (1.70), (65.58), -CF-	689.11, (1.60), (66.08), -CF-	689.02, (1.55), (60.15), -CF-
O1s BE, (FWHM), (a%), CB	531.58, (2.04), (0.84), -H/H_2_O	–	531.94, (1.96), (1.66), -H/H_2_O	–	–	532.02, (2.15), (1.34), -H/H_2_O
-CF2-/F	0.49	0.49	0.49	0.46	0.51	0.52

a%: atomic percentage; BE: binding energy (eV); CB: chemical bond; FWHM: full width at half maximum (eV); SEM: scanning electron microscopy.

**Table 3:** Mechanical and physical properties of the PTFE membranes examined (mean ± SD).

Properties	Permamem^®^	Cytoplast^TM^ TXT-200	NeoGen^®^	Surgitime	OsseoGuard^®^ TXT	OsseoGuard^®^ NTXT	*p*
Mechanical properties							
UTS (MPa)	6.0 ± 0.7	4.3 ± 1.7	14.7 ± 2.0	4.8 ± 2.0	3.6 ± 0.4	3.8 ± 1.8	<0.001
Strain (%) ^a^	0.46 ± 0.03	0.59 ± 0.27	0.48 ± 0.15	0.54 ± 0.25	2.03 ± 0.94	1.30 ± 1.47	0.15
E ^a^	7.4 ± 1.8	5.7 ± 2.1	34.4 ± 6.6	11.7 ± 6.9	3.4 ± 0.9	18.1 ± 1.3	<0.001
Physical properties							
Roughness (µm) ^a^	2.1 ± 0.5	6.9 ± 3.6	3.2 ± 1.1	32.2 ± 15.6	6.1 ± 6.2	0.9 ± 0.3	<0.001
Density (g/cm^3^)	1.57 ± 0.06	1.48 ± 0.24	1.27 ± 0.06	1.62 ± 0.12	1.35 ± 0.06	1.51 ± 0.20	0.09
Wettability (CA)	107.6 ± 0.5	96.8 ± 0.5	107.9 ± 0.3	139.4 ± 0.6	108.7 ± 8.8	88.8 ± 5.1	<0.001

^a^: Statistical comparison performed with values on a log scale; UTS: Ultimate Tensile Strength; MPa: MegaPascals; E: Young’s Modulus; CA: contact angle.

**Table 4:** Comparison of properties between PTFE membranes with textured (indentations) and non-textured surfaces and between the two textured membranes (mean ± SD).

Properties	Non-Textured	Textured	*p*	Cytoplast^TM^ TXT-200	OsseoGuard^®^-TXT	*p*
UTS (MPa)	7.3 ± 4.7	4.0 ± 1.2	0.11	4.3 ± 1.7	3.6 ± 0.4	0.53
Strain (%) ^a^	0.70 ± 0.74	1.31 ± 1.00	0.99	0.59 ± 0.27	2.04 ± 0.94	0.04
E ^a^	17.9 ± 11.5	4.7 ± 2.0	0.008	5.7 ± 2.1	43.4 ± 0.9	0.14
Roughness (µm) ^a^	9.6 ± 15.2	6.5 ± 4.9	0.23	6.9 ± 3.6	6.1 ± 6.2	0.20
Density (g/cm^3^)	1.49 ± 0.18	1.42 ± 0.18	0.40	1.48 ± 0.24	1.35 ± 0.06	0.41
Wettability (CA)	110.9 ± 18.4	102.7 ± 8.6	<0.001	96.8 ± 0.5	108.7 ± 8.7	<0.001

^a^ Statistical comparison performed with values on a log scale. Abbreviations see Table 3.

**Table 5:** Descriptive statistics for preplanned surface-type comparison groups.

Variable	Combined Groups of Membranes		
	Textured	Non-Textured	*p*-Value
Material volume (μm^3^)			
Median	1,310,736	1,000,850	0.98
IQR	514,594	309,093	
Defect volume (μm^3^)			
Median	222,128	208,874	0.13
IQR	954,764	999,934	
Defect volume ratio (%)			
Median	13,705	1197	0.011
IQR	109.523	195.569	
Thickness (μm)			
Median	216.69	218.32	0.27
IQR	38.41	17.25	

## Data Availability

Not applicable.
